# Progress in Somatic Embryogenesis of Japanese Pines

**DOI:** 10.3389/fpls.2019.00031

**Published:** 2019-01-28

**Authors:** Tsuyoshi E. Maruyama, Yoshihisa Hosoi

**Affiliations:** Department of Forest Molecular Genetics and Biotechnology, Forestry and Forest Products Research Institute, Tsukuba, Japan

**Keywords:** clonal propagation, cryopreservation, embryogenic cultures, gellan gum, liquid culture, polyethylene glycol, protoplasts, somatic embryo desiccation

## Abstract

Somatic embryogenesis (SE) in not only one of the most promising techniques for mass propagation of selected trees, but also is a valuable tool for basic research studies in cell biology and genetic engineering, and it allows the long-term *ex situ* conservation of genetic resources by cryopreservation techniques. This review reports the most recent progress in SE, protoplast culture, and cryopreservation of four important Japanese pines (*Pinus thunbergii, Pinus densiflora, Pinus armandii* var. *amamiana*, and *Pinus luchuensis*). Induction of embryogenic tissues (ET), embryogenic culture maintenance/proliferation, production of somatic embryos, germination, and conversion to plants are described focusing on the protocols most commonly reported for plant production in *Pinus* species through to SE.

## Introduction

Since somatic embryogenesis (SE) was developed for spruce species in the late 1980s, this technology has expanded to many conifer and hardwood species. SE exhibits potential for efficient and economical mass production of clonal planting stock (Becwar, 1993). Additionally, embryogenic cells can be long-term stored in liquid nitrogen without changing its original juvenility potential (Park et al., 1998; Find et al., 2014). Somatic embryo production and cryopreservation from embryogenic cells of several important species have been conducted (Sutton, 2002). Furthermore, SE is an ideal system for synthetic seed technology (Reddy et al., 2012), genetic engineering (Henderson and Walter, 2006), and plant cell biology studies (Fowke et al., 1995). Therefore, numerous studies on SE concentrate on the development and improvement of methods for mass propagation in conifers (Tautorus et al., 1991; Jain et al., 1995b; Klimaszewska and Cyr, 2002; Stasolla et al., 2002; Jain and Gupta, 2005; Klimaszewska et al., 2007). However, for many species, *in vitro* clonal propagation is still difficult or achieved at low efficiency levels (Bonga et al., 2010). The low initiation frequency of SE, low number of embryos generated, and low frequency of plant conversion are the leading problems that hamper the efficient large-scale production of some species and limit their widespread practical uses. The efficient production of high-quality somatic embryos is the most important criterion for using SE protocols in commercial propagation and tree improvement programs (Maruyama and Hosoi, 2016).

*Pinus thunbergii* Parl. (Japanese black pine), *Pinus densiflora* Zieb. *et* Zucc. (Japanese red pine), *Pinus armandii* Franch. var. *amamiana* (Koidz.) Hatusima (Yakutanegoyou), and *Pinus luchuensis* Mayr. (Ryukyu pine) are important native species in Japan used for reforestation (Maruyama and Hosoi, 2014). Japanese black pine is also important for protection of the coastal areas, and Japanese red pine is the principal host species of the prized “matsutake mushroom” (Maruyama and Hosoi, 2016). Ryukyu pine is valued for construction of houses and furnishings (Hosoi and Maruyama, 2012). Yakutanegoyou, an over-harvested species, was traditionally used for construction of houses and canoes; this species is now endangered and has estimated numbers of living trees of 100 and 1000–1500 in the natural stands of Yakushima and Tanegashima Islands, respectively (Maruyama et al., 2007). The populations of these four pines have notably declined due to pine wilt disease, caused by the pinewood nematode *Bursaphelenchus xylophilus* (Maruyama and Hosoi, 2014). Since its introduction into Japan from North America, the pine wilt disease has rapidly spread to China, Korea, and Taiwan (Togashi and Shigesada, 2006) and also has devastated pine forests in Portugal, Spain and other European countries (Mota et al., 1999; Nunes da Silva et al., 2015). Therefore, it is essential to establish a practical and effective plant regeneration method for mass propagation of resistant clones (Maruyama and Hosoi, 2016).

This review describes progress in SE of four species of Japanese pines (JPs) over the last decade, focusing on the two protocols most commonly reported for plant production in *Pinus* species through to SE (Maruyama and Hosoi, 2016). Somatic plant regeneration from maturation protocols using polyethylene glycol (PEG) or gellan gum (GG) at a high concentration are compared, and the positive effect of somatic embryo desiccation after PEG-mediated maturation is emphasized (Maruyama and Hosoi, 2012). In addition, protoplast culture and cryopreservation approaches from embryogenic tissues (ET) are also reported.

## General Consideration on SE in Pines

After the first report on SE in *Pinus taeda* by Gupta and Durzan (1987), many studies on SE in other pine species have been reported (Bajaj, 1991; Tautorus et al., 1991; Gupta and Grob, 1995; Jain et al., 1995b; Morohoshi and Komamine, 2001; Klimaszewska and Cyr, 2002; Park et al., 2006, 2016; Klimaszewska et al., 2007; Maruyama and Hosoi, 2014; Jain and Gupta, 2018). However, despite the optimization of protocols in some species of industrial importance such as *P. taeda, Pinus radiata, Pinus pinaster*, and *Pinus strobus*, in many other species, e.g., for JPs, the SE protocols are not yet well refined. Bottlenecks related to a low frequency of SE initiation, a low production of somatic embryos, or a low frequency of germination and conversion are the main impediments that must be solved for practical applications.

SE is a complex multistage process, in which each stage represents different approaches, and these are strongly dependent on the result of the previous stage (Klimaszewska and Cyr, 2002). In general, the SE process in *Pinus* species can be divided into the following stages:

(1) Induction of ET: generally, from seed explants cultured in darkness on semi-solid medium containing a combination of auxin and cytokinin. The use of whole megagametophyte containing developing immature zygotic embryos has become the most popular method for induction of ET in pine species (Klimaszewska et al., 2007). The frequency of ET induction, calculated from the number of cell lines with stable proliferation capacity, strongly depends on the developmental stage of explants and genotype.
(2) Proliferation of ET: maintenance and proliferation of induced ET by continuous subcultures in darkness onto a fresh semi-solid medium (usually of the similar composition as the used for SE initiation) at 2- to 3-week intervals. For maintenance, the ET may be cryopreserved (Park et al., 1998). For fast proliferation, the ET may be culture in liquid medium (Maruyama et al., 2005b; Pullman, 2018).
(3) Maturation of somatic embryos: development of early (immature) somatic embryos into cotyledonary (mature) somatic embryos by culture of ET on semi-solid maturation medium, typically containing abscisic acid (ABA) to replace auxin and cytokinin used for induction of ET and proliferation stage, and supplemented with an osmotic regulator agent (generally PEG) or a high concentration of gelling agent (generally GG) to reduce water availability to the cultures. After transfer onto maturation medium, ET gradually develops an individual and compact mass (proembryo), which follows additional stages of development until becoming mature cotyledonary embryos. The development and maturation patterns of somatic embryos in pines are described by several authors (Smith, 1996; Becwar et al., 1988; Lelu et al., 1999; Pullman et al., 2003).
(4) Post-maturation treatment of somatic embryos: partial desiccation of somatic embryos prior to germination after maturation onto PEG-supplemented medium, is a conventional process to enhance and synchronize somatic embryo germination in pines and spruce species. The aim of partial desiccation of somatic embryos is to reduce the water content and/or to complete the maturation process (Roberts et al., 1990). However, partial desiccation is not necessary if the water content of somatic embryos is adequately low at the end of the maturation period, e.g., when somatic embryos are matured on media containing high concentrations of GG (Klimaszewska and Cyr, 2002; Maruyama and Hosoi, 2016).
(5) Germination and plant conversion: cotyledonary somatic embryos are generally germinated onto plates containing semi-solid hormone-free medium under illumination conditions. Germination is recorded after the emergence of root, and plant conversion when both root and epicotyl develop from somatic embryos to become into plantlets, after usually 6 and 12 weeks of culture, respectively (Maruyama and Hosoi, 2014).
(6) Growth *in vitro* and acclimatization of somatic plants: development of plantlets *in vitro* for several weeks before *ex vitro* acclimatization is usually performed using culture flasks or boxes, under same conditions as the used for germination and plant conversion. Then, developed somatic plants are transplant into pots or containers filled with substrate and acclimatized usually under high relative humidity during the first 2 weeks of the process. Subsequently, the relative humidity is gradually reduced until the somatic plants acclimatize with the external conditions (Maruyama and Hosoi, 2018).

## History of SE in JPs

The SE and plant regeneration in JPs was first described in *P. thunbergii* by Ishii and Maruyama (2000), followed by other reports (Hosoi and Ishii, 2001; Ishii et al., 2001; Taniguchi, 2001). Subsequent studies reported on SE in JPs described the high maturation rates of somatic embryos, nevertheless, the germination and plant conversion frequencies remained low (Maruyama et al., 2005a,b, 2007; Shoji et al., 2006). After 5 years, Maruyama and Hosoi (2012) developed an improved regeneration protocol for JPs based on partial desiccation treatment after maturation of somatic embryos. This treatment not only notably improved the germination rates, but also accelerated and synchronized the germination period. On the other hand, as reported in other conifers (Klimaszewska and Cyr, 2002; Kim and Moon, 2007a; Lelu-Walter and Pãques, 2009), maturation protocol that involves the water availability reduction for culture cells by increasing the gel strength of the medium with a high concentration of GG also improved embryo germination and conversion in JPs (Maruyama and Hosoi, 2016).

## Initial Plant Material for Induction of ET

Selection of the appropriate explant is one of the most critical factors for successful induction of ET in conifers (Tautorus et al., 1991). At present, the greatest success has been achieved with immature zygotic embryos (Cairney and Pullman, 2007). Becwar et al. (1988) reported that embryo development at the cotyledonary and precotyledonary stage is the best for induction of ET in *Picea* and *Pinus* species, respectively. SE from mature seeds was reported in several *Picea* species (Tremblay, 1990; Harry and Thorpe, 1991). Nevertheless, for *Pinus* species, the low induction frequencies reported using mature seed explants indicate the difficulty to be considered for practical application (Klimaszewska et al., 2007). Although the use of mature stored seeds has the advantage of providing explants over a longer period (Tautorus et al., 1991), control of ET induction from mature embryos remains difficult for many forest tree species. Research over the past 20 years indicates that induction of ET in pines is initiated most efficiently when using entire megagametophytes containing immature zygotic embryos (Finer et al., 1989; Becwar et al., 1991; Klimaszewska et al., 2007). However, the period when the explants are highly responsive to induce ET is limited to a few weeks per year. In addition to the restricted time for ET induction, the use of immature seed explants may produce ET which is integrated of various paternal genotypes as reported in loblolly pine (Becwar et al., 1991). This study revealed that ET can be initiated from subordinated embryos genetically different to the dominant embryo, but the frequency of appearance and the effect on clonal propagation in pine species remain unclear (Klimaszewska et al., 2007). In open-pollinated cones, the criteria for the optimal time collection of explants cannot be easily generalized due to the difficulty in defining the precise time of fertilization that can vary with climate conditions and location of trees (Maruyama et al., 2002). Moreover, because all crosses do not develop simultaneously, it is common to observe variation in the developmental stage of embryos among trees, between the cones of the same tree, and even within the same cone (Maruyama et al., 2000). Although the monitoring of the developmental stage of individual embryos is the most suitable method for defining the best time for seed collection, this method is impractical when whole megagametophytes are used as explants (Maruyama and Hosoi, 2014). On the other hand, recently reports for *P. radiata* indicated that when megagametophytic tissues become necrotic, they can have a negative influence on zygotic embryo, due to the exudation of chemical substances that inhibit the development of embryo tissues to initiate SE (Hargreaves et al., 2009, 2011). These authors reported that, the overall average initiation rate increased to 55% (average of 70% per family) when excised zygotic embryos were used as explant. Similarly, Reeves et al. (2018) reported that using excised zygotic embryo was superior over using whole megagametophytes for ET induction in *Pseudotsuga menziesii*. These recent reports suggest that the superiority attributed to the whole megagametophyte as explant for induction of ET is relatively questionable. However, as far as we know to date, excepting *P. radiata*, there are no related reports for other pine species regarding to superiority of excised zygotic embryo over using whole megagametophyte as explants for induction of ET.

In fact, the low induction rate of ET is one of the critical factors that should be resolved for practical uses (Maruyama and Hosoi, 2014). Although seed genotypes and culture methods may affect induction of ET (Miguel et al., 2004; MacKay et al., 2006), the proper stage of embryo development is apparently the most important factor (Becwar et al., 1988). In many *Pinus* species the optimal developmental stage of the embryo is reported as the time after fertilization or seed collection date (Becwar et al., 1990). However, the low induction frequencies in several species suggest considerable variations in embryo developmental stages, even when collected at the same time. Improving the induction frequencies of ET is important to develop varietal lines and for the management of genetic diversity (Hargreaves et al., 2009). Refrigeration of cones can be useful to prolong the effective period for induction of ET and may positively influence on initiation frequencies (Attree et al., 1991). Hakman and Fowke (1987) reported that 2 months of storage at 4°C was found to be best for seeds of *Picea glauca*. Montalbán et al. (2015) improved the success of ET induction in *P. radiata* by storing seeds at cold conditions for 1–3 months. Hence, cold preconditioning of initial plant materials could be an alternative to improve ET induction success in conifers.

Induction of ET from vegetative shoots or needle explants of mature trees has been achieved in other *Pinus* species (Malabadi and Nataraja, 2007; Malabadi et al., 2011). Although SE from adult vegetative material of selected trees is the best method for mass propagation of resistant clones against pine wilt disease, unfortunately until now, the reported protocols never achieved success with JPs. More efforts are needed to establish an embryogenic system from mature trees of JPs for effective breeding programs for resistance to pine wilt disease (Maruyama and Hosoi, 2018). Nowadays, the improvement programs for resistance to pine wilt disease in JPs are based mainly on the selection of plants produced by seeds or SE from controlled pollination crosses of resistant parents (resistant mother and father trees). SE from megagametophytes explants also has the potential to produce rejuvenated clones, which after the resistance selection, can serve as mother material in scion gardens for vegetative propagation of resistant plants.

## Induction and Proliferation of ET

Medium components and culture conditions are important factors affecting induction of ET. Different formulations of media are used in ET induction of pine species; and the commonly used media are MS (Murashige and Skoog, 1962), LP (Quoirin and Lepoivre, 1977), DCR (Gupta and Durzan, 1985), LV (Litvay et al., 1985), MSG (Becwar et al., 1990), and embryonal masses (EM) (Smith, 1996) in original or modified compositions. The media are commonly supplemented with L-glutamine and/or casein hydrolysate (instead of reducing the concentration of inorganic nitrogen), plant growth regulators (PGRs), sugar, and other additives, such as activated charcoal (AC) (van Winkle and Pullman, 2005), amino acids (Smith, 1996), silver nitrate (Kong and Yeung, 1994), biotin, folic acid, and pH buffer agent (Pullman et al., 2005). Although sucrose (1–3%) is the most popular carbon source, maltose was reported more effective than sucrose, glucose, or fructose for ET initiation in *Pinus nigra* (Salajová and Salaj, 2005). GG is the most frequently used gelling agent to solidify a medium. 2,4-Dichlorophenoxyacetic acid (2,4-D) and 6-benzylaminopurine (BA) are the most common PGRs used for SE initiation and proliferation of ET; occasionally, for SE initiation, naphthaleneacetic acid (NAA) is used instead of 2,4-D (Gupta, 2014), and *N*-(2-chloro-4-pyridyl)-*N*’-phenylurea (CPPU) is used as sole PGR in the medium (Park et al., 2006). Supplement of other PGRs, such brassinolides, triacontanol, and salicylic acid, can improve SE initiation rates (Malabadi et al., 2011). Pullman et al. (2008) reported improvements in ET induction by supplementing the medium with D-xylose and D-*chiro*-inositol. In general, the cultures are kept in darkness at 23–26°C for induction of ET and ET maintenance and proliferation.

ET induction of JPs was initiated from whole megagametophytes containing immature zygotic embryos (Maruyama et al., 2005a,b, 2007; Hosoi and Maruyama, 2012). Seeds were disinfected with 1–2% sodium hypochlorite solution for 15–20 min (Maruyama and Hosoi, 2014). Then, aseptically removed megagametophytes explants were cultured on the initiation medium (Maruyama et al., 2000) containing 10 g l^-1^ sucrose, 0–10 μM 2,4-D, 0–5 μM BA, 0.5 g l^-1^ casein hydrolysate, 1 g l^-1^ L-glutamine, 0–2 g l^-1^ AC, and 3 g l^-1^ GG (Gelrite^®^; Wako Pure Chemical, Osaka, Japan). The pH of the medium was adjusted to 5.8 before sterilization; the plates were sealed with Parafilm^®^and kept in darkness at 25°C (Maruyama and Hosoi, 2016). In general, the extrusion of ET occurred 2–6 weeks after culture, and the proliferation of ET became obvious after 4–8 weeks of culture when EM increased (Maruyama and Hosoi, 2014). The induction frequency on the medium supplemented with PGRs varied from 1.0 to 2.8%, with an overall average of 2.3% (Maruyama et al., 2005a,b, 2007; Hosoi and Maruyama, 2012), which was comparable with previous results for *P. densiflora* (up to 2.3%, Kim and Moon, 2014), *P. nigra* (3.1%, Salajová and Salaj, 2005), *Pinus banksiana* (up to 3.9%, Park et al., 2006), *P. rigida* × *P. taeda* (up to 1.1%, Kim and Moon, 2007b), and *P. lambertiana* (1–3%, Gupta, 1995). These results were inconsistent with the high induction rates reported for *P. radiata* (44–93%, Hargreaves et al., 2011), *P. taeda* (up to 79%, Gupta, 2014), *P. strobus* (54%, Finer et al., 1989), *Pinus sylvestris* (up to 30%, Aronen et al., 2009), and *P. pinaster* (up to 75%, Park et al., 2006).

Induction of ET was also possible on medium without exogenous PGRs as described for *P. radiata* (Smith, 1996), *P. sylvestris*, and *P. pinaster* (Lelu et al., 1999) and for other *Cupressaceae* trees, such as Japanese cedar (Maruyama et al., 2000), Hinoki cypress (Maruyama et al., 2005c), Atlantic cedar (Ahn et al., 2017), and Oriental thuja (Ahn and Choi, 2017). The reported results suggest that the addition of exogenous PGRs is not necessary if the explants are in the appropriate developmental stage (Maruyama et al., 2007). However, in JPs, the induction frequencies were lower than that achieved on medium supplemented with PGRs; approximately 64% of families (18 out of 28) responded in medium containing PGRs, and only 48% of families (12 out of 25) responded in the medium without PGRs (Maruyama et al., 2005a,b, 2007). Differences in ET induction frequencies observed among families (one family means seeds collected from one mother tree) in other pine species indicate that induction success is strongly dependent on the genotype of the explants (Niskanen et al., 2004; Hargreaves et al., 2009). In JPs, a total of 58 from 3045 megagametophytes tested (1.9%) extruded ET, and the number of explants with proliferating ET decreased, and only 1.3% of the explants continued to proliferate and established embryogenic cultures (Maruyama et al., 2005a,b, 2007; Hosoi and Maruyama, 2012). As reported for many species, extruded ET did not always lead to proliferation of EM that resulted in stable embryogenic lines. Kim et al. (1999) reported that only one embryogenic cell line, within 294 lines inducted in Japanese larch, had the ability to proliferate. In ET induction of *Pinus rigida* × *P. taeda*, Kim and Moon (2007b) reported that only two viable lines survived out of 52 lines produced from 11,388 explants. Studies in several pine species reported the decreased number of explants with proliferated ET from explants with initiated extrusion (Garin et al., 1998; MacKay et al., 2006). Given that extrusion is insufficient for establishment of an embryogenic line in some instances, the initial extrusion from an explant should be distinguished from stable continuous growth when assessing ET induction (Klimaszewska et al., 2007). The induction of stable ET lines should be the criterion to consider when evaluating ET induction capacity (Maruyama et al., 2007).

A recent work reported an improved ET induction rate (up to 15%) for some genotypes of *P. thunbergii* (Maruyama et al., 2016). Due to the fact that improvement was related to the genetic characteristics of some donor trees and that many recalcitrant genotypes generate a great variation among families, the selection of responsive seed genotypes by multi-year screening is necessary for improvement of ET induction frequencies. A predictable improvement in ET induction could be achieved by selection of appropriate parents (MacKay et al., 2006). Improved protocols already used for *P. radiata* (Hargreaves et al., 2011), efficiently improved the average induction rate of ET in Douglas-fir; such protocols include disinfection of whole cones (instead of seeds), modification of induction medium (GlitzB medium), and culture of zygotic embryos (instead of whole megagametophytes) (Reeves et al., 2018). Gupta (2014) reported a notable improvement of 3–4 times of the average induction rate of ET in loblolly pine by culturing transversally dissected megagametophytes (two parts, instead of whole explant) in solid initiation medium and post-addition of liquid medium containing 10 mgl^-1^ ABA after 2 weeks of culture.

Smith (1996) conducted maintenance and proliferation of ET in medium without PGRs in *P. radiata*. In the same way, Breton et al. (2005) recommended weekly subculturing of *P. pinaster* EM on maltose-containing medium without PGRs to keep embryogenic capability. However, in JPs, ET maintained in the PGR-free medium exhibited a propensity to spontaneous development going to the maturation stage, and showed a decline in proliferation capacity over time (Maruyama and Hosoi, 2014). Although induction of ET was possible in absence of exogenous PGRs, for JPs the medium supplemented with PGRs was the best for supporting the growth of embryogenic cells (Maruyama et al., 2005a,b, 2007; Hosoi and Maruyama, 2012). Proliferation was promoted by culturing ET in clumps on semi-solid proliferation medium (fresh initiation medium but containing 3 μM 2,4-D, 1 μM BA, 30 g l^-1^ sucrose, and 1.5 g l^-1^ glutamine) at 2- to 3-week intervals (Maruyama and Hosoi, 2016). Embryogenic cultures proliferated readily and depending on the lines, the fresh weight (FW) of ET increased 5–15 folds after 2–3 weeks of culture (Maruyama and Hosoi, 2014). To accelerate its proliferation, ET were cultured in conical flasks containing the same proliferation medium but without GG (Maruyama et al., 2005b).

## Maturation of Somatic Embryos

The composition of medium for maturation of somatic embryos is generally the same as for previous stages (at standard concentration) and contains ABA to replace auxin and cytokinin. Several studies on SE in conifer species have reported the importance of ABA in embryo development process (Maruyama et al., 2007); suggesting that ABA is closely related with the accumulation of carbohydrates, lipids, and proteins during the maturation process, and with the suppression of precocious germination (Kong and von Aderkas, 2007; Rai et al., 2011). Morphological studies in pro-embryogenic masses during maturation of Chinese fir somatic embryos, revealed that ABA enhanced the development of EM, and that PEG worked organizing pro-embryo structures; and that both roles were complementary during the pro-embryo formation (Zhou et al., 2017). By contrast, the application of ABA did not significantly benefit the somatic maturation of some *Cupressaceae* trees, but also on higher concentrations the cultures lowered production, became necrotic, and did not undergo further development (Lambardi et al., 1995). Bonga and von Aderkas (1993) reported that some lines of embryogenic cultures of European larch subcultured in the medium without ABA supplement over a 4-year period produced large quantity of normal somatic embryos.

The requirements of carbohydrates during the embryo maturation process can vary according to the species or even among cell lines (Tremblay and Tremblay, 1991). The efficiency of SE is strongly influenced by culture conditions, within which the supply of carbohydrates plays one of the most critical roles (Lipavská and Konrádová, 2004). Sucrose is an efficient carbohydrate in SE of *Picea* species (Iraqi and Tremblay, 2001), and its combination with a high concentration of GG is commonly used for somatic embryo maturation (Klimaszewska et al., 2007). Moreover, the mixture of sucrose with PEG was also used for somatic embryo production in Scots pine (Aronen et al., 2009). Maltose is many times reported as better carbohydrate for embryo development; and supplemented with PEG is widely utilized in a large number of conifer species (Jain et al., 1995b; Jain and Gupta, 2005). Other researchers reported on media supplemented with other carbohydrates, such as lactose, myo-inositol, sorbitol, or mannitol (Schuller and Reuther, 1993; Jain et al., 1995b; Maruyama et al., 2002). The combination of 88 mM sucrose and 175 mM sorbitol in the maturation medium solidified with 1% GG was reported as efficient to produce somatic embryos in *P. strobus* (Garin et al., 2000). Tereso et al. (2007) performed a comparative study on morphogenesis of zygotic and somatic embryo in *P. pinaster* and reported differences in maturation yield, accumulation of proteins and starch, and anatomical morphogenesis derived from different carbohydrate sources and concentrations.

For somatic embryo maturation of JPs, fresh ET were cultured in clumps or suspended in liquid medium and then poured over maturation medium (modified from Maruyama et al., 2000); containing 30–60 g l^-1^ maltose, 0–100 μM ABA, 0–2 g l^-1^ AC, amino acids (in g l^-1^: glutamine 0.75–7.3, asparagine 0.5–2.1, arginine 0.25–0.7, citrulline 0.079, ornithine 0.076, lysine 0.055, alanine 0.04, and proline 0.035), and 0–150 g l^-1^ PEG (Av. Mol. Wt.: 3000–9300; Wako Pure Chemical, Osaka, Japan) or a high concentration of GG (10 g l^-1^, without PEG) (Maruyama and Hosoi, 2016). The cotyledonary somatic embryos were clearly distinguished about 3–4 weeks after transfer of ET; and they completed their maturation mostly within about 8 weeks of culture (Maruyama and Hosoi, 2016). The supplement of PEG had a beneficial impact on maturation of somatic embryos, and this impact was even more stimulated with the supply of AC (Maruyama and Hosoi, 2016). The effect of PEG as osmoticum in culture medium may be associated to the induction of water stress, dehydration tolerance, and accumulation of some storage reserves (Roberts et al., 1990; Misra et al., 1993). At present, the application of PEG with ABA has become the most used and successful procedure for promoting embryo development in several genera of conifers (Stasolla and Yeung, 2003). Similar to JPs, the beneficial effect of AC has been widely reported in other conifer trees (Jain et al., 1995b; Maruyama et al., 2000, 2002, 2005c; Kim and Moon, 2014; Ahn et al., 2017). The positive effect of AC may be related to the capture of residual PGRs, undesirable substances, and other toxic metabolites present in the culture medium (Pan and van Staden, 1998). In contrast to the high somatic embryo productivity on PEG-supplemented media, only few cotyledonary embryos were achieved in PEG-free medium (Maruyama et al., 2005b, 2007; Hosoi and Maruyama, 2012; Maruyama and Hosoi, 2016). Typically, ET proliferation was evident in PEG-free medium, and most of them developed into structures similar to those of the (stage 1 somatic embryo) described by von Arnold and Hakman (1988).

On the other hand, notwithstanding that the overall average of embryos reported was lower than that achieved in the PEG containing media, the addition of 10 g l^-1^ GG (into maturation medium without PEG) was found to be beneficial in somatic embryo maturation of JPs (Maruyama and Hosoi, 2016). Moreover, in some genotypes, the quantity of mature embryos achieved in the PEG-free medium containing a high concentration of GG was greater than that recorded in the medium containing PEG (Maruyama, unpublished). In the same way, improved maturation protocols for various *Pinus* species have reported (Smith, 1996; Lelu et al., 1999; Percy et al., 2000; Klimaszewska et al., 2007). Despite that maturation frequencies varied among species and cell lines, maturation medium containing PEG (100 g l^-1^), maltose (30–50 g l^-1^), AC (2 g l^-1^), and ABA (100 μM), or PEG-free maturation medium supplement with 10 g l^-1^ GG were found to be appropriate for production of somatic embryos in JPs (Maruyama and Hosoi, 2014, 2016).

## Germination and Plant Conversion

Cotyledonary embryos of JPs were cultured onto germination medium (modified from proliferation medium, containing no PGRs, and supplemented with 30 g l^-1^ glucose, 0.4 g l^-1^ glutamine, 0.25 g l^-1^ arginine, 0.1 g l^-1^ proline, 2 g l^-1^ AC, and 6 g l^-1^ GG). Usually, germination (emergence of root) and plant conversion (emergence of both root and epicotyl) were recorded 6 and 12 weeks after culture at 25°C under a photon flux density of about 65 μmol m^-2^ s^-1^ with a 16 h photoperiod, respectively (Maruyama and Hosoi, 2016). For post-maturation treatment, cotyledonary embryos were harvested from PEG-mediated maturation medium and exposed to partial desiccation before transfer to germination medium as described by Maruyama and Hosoi (2012). In brief, embryos over filter paper sheets were placed into two central wells of a six-well plates and the remaining wells were filled with about 5 ml of sterile water; then plates were sealed with Parafilm^®^, and kept in the dark at 25°C for 3 weeks.

For JPs, germination and plant conversion occurred at low frequencies (an overall average of 18.4% and 14.4%, respectively) when somatic embryos matured in the medium containing PEG were directly transferred to germination medium (Maruyama and Hosoi, 2016). This result confirmed that for JPs, PEG stimulates maturation of somatic embryos but inhibits subsequent germination process, as similarly reported in *Picea abies* (Bozhkov and von Arnold, 1998). Partial desiccation of cotyledonary embryos considerably increased the germination rates and also improved the subsequent plant conversion frequencies in JPs. Germination and conversion was commonly observed after 1–2 and 4–8 weeks of culture, respectively; and the overall average frequencies of the desiccated embryos of JPs were improved by around four to five-fold compared with the control (Maruyama and Hosoi, 2016). To improve plant conversion in a number of conifers after PEG-mediated maturation protocols, cold treatment and/or partial desiccation of somatic embryos has been recommended (Roberts et al., 1990; Klimaszewska et al., 2007). In *Pinus oocarpa*, a 2 to 3-week period of desiccation was reported to be beneficial in increasing germination efficiency; by contrast, without desiccation, embryos germinated abnormally, with signs of vitrification, dying later (Lara-Chavez et al., 2011). Similarly, desiccation after PEG-mediated maturation improved the somatic embryo germination rates of interior spruce (Roberts et al., 1990), white spruce (Attree et al., 1995), hybrid larch (Lelu et al., 1995), and patula pine (Jones and van Staden, 2001). Cold treatment (stratification treatment for 16 days at 4°C in darkness on PGRs-free medium) alone or in combination with partial desiccation increased the germination percentage in slash pine (Liao and Amerson, 1995), white spruce (Pond et al., 2002), Taiwan spruce (Liao and Juan, 2015), and Fraser fir (Pullman et al., 2016).

After maturation onto PEG-free medium containing GG at a high concentration, the overall frequencies of germination and plant conversion achieved in JPs were around 80% and 78%, respectively; without application of any post-maturation treatments (Maruyama and Hosoi, 2015, 2016). Researches on other *Pinus* species reported that restricting water availability by increasing the medium gel strength improved germination and conversion rates in *P. radiata* (Smith, 1996), *P. strobus* (Klimaszewska and Smith, 1997), *P. sylvestris* (Lelu et al., 1999), *P. monticola* (Percy et al., 2000), *P. pinaster* (Lelu-Walter et al., 2006), and *P. halepensis* (Montalbán et al., 2013).

## Growth *In Vitro* and Acclimatization of Somatic Plants

The growth *in vitro* of somatic plantlets of JPs was encouraged by transferring them into culture-flasks with germination medium containing 30 g l^-1^ glucose or sucrose, 5 g l^-1^ AC, and 12 g l^-1^ agar (Wako Pure Chemical Industries, Osaka, Japan) or into Magenta^®^vessels (Sigma, St. Louis, United States) containing Florialite^®^(Nisshinbo Industries, Inc., Tokyo, Japan) fertilized with a plant food solution modified from Nagao (1983), and kept under same conditions described above for about 15–20 weeks before acclimatization. After 15 weeks of culture all somatic plantlets survived, and the best growth performance was achieved with Florialite^®^substrate fertilized with Nagao’s solution inside Magenta^®^vessels (Hosoi and Maruyama, 2012). Subsequently, somatic plants were successfully acclimatized under the conditions described by Maruyama and Hosoi (2016); and kept for about 1 year in a greenhouse before planting to the field. Although the SE-derived trees show a normal appearance, it is necessary to examine and monitor their genetic stability using molecular marker technologies. Genetic stability and good growth performance in the field are important criteria in the establishment of a practical protocol for SE (Hosoi et al., 2015).

## Liquid Culture for Somatic Embryo Production

Liquid media for culture scale-up can allow the production of many thousands, if not millions, of propagules at a time (Aitken-Christie et al., 1995). Although bioreactors were traditionally used for bacterial fermentation or for the secondary metabolite production (Heyerdahl et al., 1995), they are nowadays also used in the mass propagation of many plant species (Leathers et al., 1995). As an example for scale-up in conifers, over 10,000 of *P. menziesii* somatic embryos were produced from a single tissue culture flask, usually in a time range from 8 to 12 weeks (Gupta and Timmis, 2005); these authors emphasize the importance of scale-up and automation to reduce the labor costs of *in vitro* propagation.

Liquid culture conditions for scale-up production were examined to enhance the somatic embryo productivity in JPs (Maruyama et al., 2016). Based on the developed protocols for solid culture, embryogenic cells were cultured using tissue culture flasks on rotary shakers or bioreactors in immersed liquid system. ET can easily proliferate in liquid medium with the equivalent composition used for solid culture. For somatic embryo production, liquid media with modifications from solid culture protocols were used; such modifications include changes in medium components, supplement of granular AC instead of powder type, and addition of the ethylene action inhibitor to the medium (K-20C, silver thiosulfate as principal active component) (Chrysal-Japan Co., Osaka, Japan). Depending on the culture conditions, the maturation of somatic embryos was observed 6–12 weeks after culture in the liquid medium. However, somatic embryo maturation was strictly dependent on cell genotype and initial culture density of the embryogenic cells (Maruyama et al., 2016). Additionally, the somatic embryo maturation was remarkably improved by supplementing K-20C. Using a medium supplemented with 2000- to 4000-fold dilution of K-20C increased the productivity by 1.5- to 3-fold compared with the untreated control (Maruyama et al., 2016). The effect of ethylene and ethylene action inhibitors on SE in conifers were reported for white spruce (Kong and Yeung, 1994), black spruce (El Meskaoui and Tremblay, 2001), hybrid larch (Saly et al., 2002), and Scots pine (Lu et al., 2011). When cotyledonary embryos of *P. thunbergii* from liquid medium were cultured onto germination medium after partial desiccation, they germinated similar to those induced in a solid medium; and showed normal morphological features after *ex vitro* acclimatization (Maruyama et al., 2016). Several thousands of somatic plants were produced by liquid culture, and a sample of them was planted in the field. Although the protocol has not been optimized, research on liquid culture conditions for JPs is currently in progress (Maruyama, unpublished). The improvement of this technology for its practical use should be conducted as soon as possible to accelerate the large-scale production of nematode-resistant clones in breeding programs.

## Protoplast Culture From ET

Regeneration from protoplasts that can form somatic plants is important for studies on cell division, cell cycle, and morphogenesis and is an effective tool for genetic modification by direct DNA transfer by electroporation, liposome fusion, and microinjection techniques (Thorpe, 2007). These techniques have gained increasing attention to develop a genome editing technology that does not contain foreign genes by direct introduction of expression vectors, such as CRISPR/Cas9 and sgRNA-Cas9 protein complexes, to protoplasts. After the first report on plant regeneration of tobacco from protoplasts (Takebe et al., 1971), tremendous progress has been reported in other important commercial crops (Bajaj, 1989). For conifer species, the first success on plant regeneration from ET-derived protoplasts was reported for white spruce (Attree et al., 1989) and hybrid larch (Klimaszewska, 1989). Protoplast isolation and culture were then reported for a number of conifer trees (Kirby et al., 1989; Lainé and David, 1990; Becwar, 1993; Bekkaoui et al., 1995; Jain et al., 1995a; Maruyama et al., 2002).

For JPs, a simple method for isolation and culture of protoplasts from ET was determined by experiments with combinations of enzymes and PGRs (Hosoi and Maruyama, 2011; Hosoi and Maruyama, 2018). The described method is as follows: approximately 5–10 g FW of ET cultured at 2-week intervals was transferred to 40 ml of an enzymatic mixture containing 1–1.5% cellulase Onozuka RS (Yakult Pharmaceutical Industry Co., Ltd., Tokyo, Japan) and 0.1–0.2% Pectolyase Y-23 (Kyowa Hakko Kirin Co., Ltd., Tokyo, Japan) in 0.6 M mannitol. The enzymatic mixture containing ET was placed in a 100 ml culture flask and incubated for 6 h in the dark at 25°C. The resulting suspension was filtered through a 30–40 μm nylon strainer to remove undigested cell clumps and debris and collected to a 50 ml centrifuge tube. The material was centrifuged at 60 × *g* for 3 min for removal of the enzyme. After centrifugation, the supernatant containing enzyme was discarded, and the protoplast pellet was re-suspended into 45 ml of 0.6 M mannitol solution in 50 ml centrifuge tube. The protoplast suspension was washed by centrifugation at 60 × *g* for 3 min. The operation was repeated twice for purification of the protoplasts. Yields of protoplasts were determined using a double-chamber hemocytometer (Improved Neubauer). The viability of the protoplasts was assessed by uptake and cleavage of fluorescein diacetate (FDA). The protoplasts were cultured at a density of 2 × 10^2^ to 5 × 10^3^ ml^-1^ into a 96-well plate (BD FALCON No.353072; Becton Dickinson and Company, Franklin Lakes, NJ, United States) containing 50 μl of liquid 1/2 EM medium, which contained 0.6 M mannitol supplemented with various combinations of 2,4-D (0.1, 0.3, 1, 3, 10, and 30 μM) and BA (0, 1, 3, and 10 μM); and the plates containing the protoplast culture in the wells were added with sterile distilled water in outspaces among the wells to maintain a high relative humidity, sealed with Parafilm^®^, and kept in the dark at 25°C.

The viability examination by FDA staining showed that the survival rate of protoplasts ranged from 60 to 100% depending on species or cell lines and experiment conditions. This result indicated that an enzyme solution containing 1–1.5% cellulase Onozuka RS, 0.1–0.2% pectolyase Y-23, and 0.6 M mannitol was effective for protoplast isolation from embryogenic material of JPs (Hosoi and Maruyama, 2011). Although the cell line and culture condition had a strong influence on protoplast production, the achieved yields were within 1.5 × 10^4^ to 5 × 10^5^ protoplasts/g FW and the size of the isolated protoplasts varied from about 20 to 60 μm in diameter (Hosoi and Maruyama, 2018). Protoplast size probably depends on the origin of the starting material; that is, small protoplasts possibly originated from embryonic cells, whereas large protoplasts were derived from suspensor cells (David et al., 1995). The protoplast yield achieved in the experiments for JPs was consistent with the reported yields from ET of *P. glauca* (1.2–4.8 × 10^5^ protoplasts/g FW, Attree et al., 1989), *P. banksiana* (0.7–5 × 10^5^ protoplasts/g FW, Tautorus et al., 1990), *P. sylvestris* (2 × 10^5^ protoplasts/g FW, Hohtola and Kvist, 1991), and *Pinus caribaea* (6.8–9.7 × 10^4^ protoplasts/g FW, David et al., 1995). In JPs, cell division and colony formation were mostly observed after 3–5 and 7–10 days of culture, respectively. Colony formation was achieved for all 24 combinations of PGRs, and the efficiency peaked in the medium with 3–10 μM 2,4-D and 1–3 μM BA (Hosoi and Maruyama, 2011, 2018). After 5–7 weeks of culture, the protoplast-derived cell suspensions were transferred to the liquid medium with a low mannitol concentration of 0.2 M. After approximately 2 weeks of culture, the proliferating cells were transferred to plates containing solid medium without mannitol and cultured at 2-week intervals for ET proliferation. For somatic embryo production and plant regeneration, the protoplast-derived cells were treated as the original ET initiated from the zygotic explants.

## Cryopreservation of ET

Although the mechanism of the loss of embryogenic potential in plants remains unknown, the ability of many embryogenic lines to form embryos decreased or is lost over time depending on the genotype. In JPs, ET can usually be maintained and proliferated for many years without any apparent morphological variation; however, most of them lose their embryogenic potential after 1 or 2 years of culture or few months after ET initiation in some instances. Breton et al. (2006) reported that for maritime pine, the maturation ability of ET was lost in less than 10 months after SE initiation. Cryopreservation is important because it can allow: (1) maintain a lot of somatic embryogenic lines, (2) provide time for data gathering regarding performance on individual lines, and (3) do mass propagation of selected lines. Given that efficient maturation and high-quality somatic plant conversion are the most important criteria for large-scale production, the time between ET induction and cryopreservation should be minimized to retain the original embryogenic potential of the cells. Long-term storage of ET by cryopreservation is a conventional and essential procedure in SE programs (Klimaszewska et al., 2007). Cryopreservation of ET permits stable maintenance of germplasm, does not change the genetic make-up, and does not induce loss of juvenility until the evaluation of the clonal progeny in the field (Tautorus et al., 1991; Park et al., 1998; Find et al., 2014).

Cryopreservation research on woody plants started with the study on cryotolerance of twig materials (Sakai, 1960). The cryopreservation of conifers was first reported in Norway spruce and loblolly pine (Gupta et al., 1987) and in white spruce (Kartha et al., 1988). Conventional cryopreservation techniques generally involve slow cooling (0.5–2.0°C min^-1^) to usually around -40°C prior to rapid immersion in LN; these techniques are basically the most important protocols but require the use of expensive and sophisticated controlled equipment and a complicated cryoprotective procedure (Sakai, 1995). After the development of cryoprotective PVS2 solution (Sakai et al., 1990), simplified cryopreservation protocols were successfully reported in a large number of temperate and tropical plant species. The PVS2 cryoprotective solution has become the breakthrough for plant vitrification research as the starting point of new vitrification-based freezing techniques (Engelman, 2004). Vitrification-based procedures by direct or slow cooling prior to plunging in LN are the most reported methods for cryopreservation of ET in conifers. Reviews on cryopreservation and recovery protocols for embryogenic cultures of several gymnosperms were reported by Charest and Klimaszewska (1995), Cyr (1999), Häggman et al. (2000), Gupta et al. (2005), Klimaszewska et al. (2007), and Li et al. (2018). Single reports on successful cryopreservation in pines, were reported in *P. taeda* (Gupta et al., 1987), *P. caribaea* (Lainé et al., 1992), *P. radiata* (Hargreaves and Smith, 1992), *P. sylvestris* (Latutrie and Aronen, 2013), *P. monticola* (Percy et al., 2000), *P. patula* (Ford et al., 2000), *P. pinaster* (Marum et al., 2004), *P. roxburghii* (Malabadi and Nataraja, 2006), and *P. nigra* (Salaj et al., 2012).

Embryogenic cultures of JPs were cryopreserved by three protocols, as described elsewhere: (1) Hargreaves and Smith (1992), (2) Sakai et al. (1990), and Sakai et al. (1991). Suspensions of about 10-day-old cultured ET were used. For subsequent growth recovery after cryopreservation, ET was cultured in liquid 1/2 EM medium (Maruyama et al., 2000) supplemented with 10 μM 2,4-D and 3 μM BAP in darkness at 25°C on rotatory shaker or onto same medium solidified with 3 g l^-1^ GG by plating the thawed embryogenic cells on filter paper disk over the medium (Maruyama et al., 2003, 2006). Growth recovery and morphological characteristics of the cryopreserved embryogenic cells were observed using an inverted and epifluorescence microscope (Nikon Optiphot, Tokyo, Japan) under U-excitation fluorescence light after staining with 4′,6-diamidino-2-phenylindole (DAPI) as described by Maruyama et al. (2003).

Although either of the freezing methods tested allowed the cryopreservation of JP embryogenic cells, the reported survival rates varied from 0 to 67%. The survival rates were strongly influenced by cell genotype (Maruyama et al., 2006). Genotypic variations in cryotolerance were reported among different genotypes of *Abies nordmanniana* (Nørgaard et al., 1993), *Picea glauca engelmanni* complex (Cyr et al., 1994), *P. nigra* (Salaj et al., 2012), and *P. sylvestris* (Latutrie and Aronen, 2013). Within 1–2 weeks of culturing, evident growth of the thawed cells was not observed. Almost all vacuolated suspensor cells showing a disrupt appearance were killed, and only the surviving embryonal head cells showed a normal appearance. This finding was similar to that reported in cryopreserved embryogenic cells of Norway spruce and loblolly pine (Gupta et al., 1987), white spruce (Kartha et al., 1988), and Sitka spruce (Kristensen et al., 1994). Similarly, as described for Japanese cedar (Maruyama et al., 2000) and Sawara cypress (Maruyama et al., 2003), the formation of new suspensor cells from surviving head cells was detected about 2–3 weeks after thawing; and about 4 weeks after culturing, an evident proliferation of cells was achieved in JPs; however, the growth rate was slower than that of cells not subjected to cryopreservation (Maruyama et al., 2006). The slow speed growth of the cryopreserved cells may be attributed to the decline in the proliferation capacity of cells due to stress of freezing or/and due to a possible damage caused by cryoprotectants (Maruyama et al., 2003). Similar results concerning to the slow speed growth of the cryopreserved embryogenic cells was reported by Gupta et al. (1987) for *P. abies* and *P. taeda*. These authors indicated that within 35 days of culturing, the evident growth of the cryopreserved embryogenic cells was not recorded, and that these cells showed normal growth rates after three subculture routines. By contrast, Touchell et al. (2002) reported that the growth of the cryopreserved ET of *Picea mariana* was the same as that of the untreated controls 10–14 days after thawing. Similarly, consistent proliferation from some cryopreserved embryogenic lines of *P. pinaster* was observed after a 2-week culture period (Lelu-Walter et al., 2006).

Studies aimed to improve the growth recovery of embryogenic cells after cryopreservation, indicated that, the initial cell density is a critical factor in the cell regrowth of *P. abies* (Find et al., 1998) and *P. pinaster* (Marum et al., 2004). In the same way, a nurse culture technique was reported to be beneficial to improve post-thaw recovery of cryopreserved *P. radiata* ET; the nurse cells (non-cryopreserved actively growing ET) presumably enhance the recovery of frozen cells by helping in the removal of remaining cryoprotectants and absorbing compounds released by damaged cells (Hargreaves et al., 2002). Utilization of ET with a high growth activity and retaining its regenerative capacity are critical factors affecting the success of growth recovery after cryopreservation (Maruyama et al., 2006). The importance of selecting actively growing cell lines to ensure optimal ET recovery after cryopreservation was emphasized in the studies on *P. caribaea* (Lainé et al., 1992) and *P. radiata* (Hargreaves and Smith, 1992). Find et al. (1998) reported that the period of culture prior to cryopreservation greatly affected the regrowth of *P. abies* and *Picea sitchensis* cryopreserved cultures. The cryopreserved cells of JPs showed normal growth rate, which resembled that of non-cryopreserved cells, mostly after two to three subculture routines. Similar pattern of growth was reported between frozen and unfrozen embryogenic cells in other Japanese conifers (Maruyama et al., 2000, 2003). Thereafter, cryopreserved cells were transferred to the maturation medium and treated as the original unfrozen ones. Somatic embryo production and regenerated plants from the cryopreserved-derived embryogenic cells of *P. thunbergii, P. densiflora*, and *P. armandi* var. *amamaina* were similar to the non-cryopreserved ET (Maruyama et al., 2006). However, it is extremely important to examine and monitor the genetic stability during the cryogenic storage of embryogenic lines. Further works are required to improve the cryopreservation system in JPs that should include long-term monitoring of somatic plants from a large number of cryopreserved lines.

## Concluding Remarks

Somatic embryogenesis has become a method not only for mass propagation but also a stepping stone for development of other biotechnologies for manufacturing of new products in forestry (Klimaszewska et al., 2011). Nevertheless, although propagation by SE has been developed for various conifers, the low induction frequency of ET and the low plant conversion rate are the limiting factors for widespread practical uses in many species (Maruyama and Hosoi, 2016). The efficient maturation of a large number of genotypes and the production of somatic plants with a high field performance are the most important criteria for using SE protocols not only in commercial production and improvement programs but also as a valuable tool for studies of molecular, genetic, and physiological processes involved in cell cycle, organ formation, and gene expression.

**FIGURE 1:**
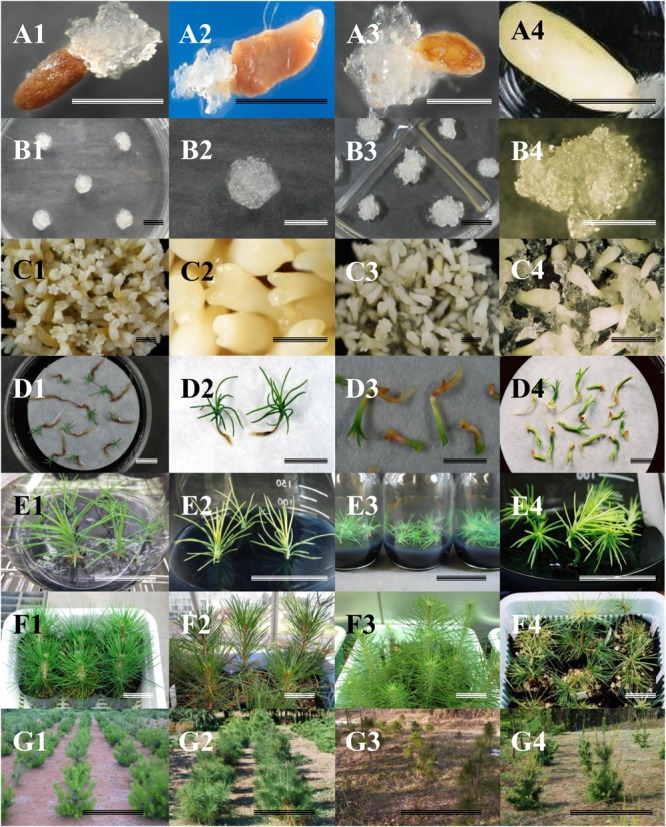
Somatic embryogenesis in Japanese pines. **(A)** Induction of embryogenic tissues. **(B)** Proliferation of embryogenic tissues. **(C)** Maturation of somatic embryos. **(D)** Germination. **(E)** Plant conversion. **(F)** Acclimatized plants. **(G)** Somatic plants growing in the field. **1**: *Pinus thunbergii*, **2**: *P. densiflora*, **3**: *P. luchuensis*, **4**: *P. armandii* var. *amamiana. Bars:* 1 cm **(A–D)**, 5 cm **(E–F)**, 1 m **(G)**.

As shown in Figure 1, an enhanced propagation protocol via SE for JPs has been achieved (Maruyama and Hosoi, 2014). Somatic embryo production and plant conversion were achieved by the two protocols most commonly used for plant production in pine species through to SE (Maruyama and Hosoi, 2016). The present work also describes a simple protocol for protoplast culture (Hosoi and Maruyama, 2011, 2018) and cryopreservation from ET (Maruyama et al., 2006). Although this improvement represents an advance in the propagation of these species, it is necessary to refine the protocol to enhance ET induction frequencies, as well as to improve the productivity of high quality embryos in liquid medium. Also, the examination and monitoring of genetic stability during the cryogenic storage of embryogenic lines, as well as of the somatic embryos obtained, is extremely important. The genetic stability of the embryogenic lines is essential in commercial propagation and tree improvement programs and, above all, a basic requirement for deployment strategies in multivarietal forestry.

In addition, even more efforts are necessary to develop an efficient methodology for induction of ET from vegetative material of adult trees. At present, attempts by a number of research groups has produced very limited success (Klimaszewska et al., 2011). Publications by Malabadi and collaborators described the successful induction of ET from adult trees of several tropical and subtropical pines (Malabadi et al., 2011; Teixeira da Silva and Malabadi, 2012). Basing on these reported protocols, considerable efforts to induce ET from adult vegetative material of JPs have been made; however, thus far, never positive results have achieved (Maruyama, unpublished). International efforts to induce ET in adult pine trees have been reported for six economically important species (*P. pinaster, P. sylvestris, P. radiata, P. patula, P. strobus*, and *P. contorta*). Despite multi-year experiments by various research groups in five countries (France, Canada, Spain, New Zealand, and Finland), induction of ET was induced only in *P. sylvestris*, but the embryo maturation frequency was too low and the harvested somatic embryos failed to grow into plants. This multi-national study suggests that the positive results obtained in the above methods reported by Malabadi and collaborators can be unrepeatable with other *Pinus* species, especially with regard to the high induction frequencies of ET (Trontin et al., 2016). An effective and practically reproducible technique to produce somatic plants from adult vegetative material must be developed for rapid acceleration of many tree improvement programs.

## Author Contributions

TM and YH conceived, designed and performed the experiments, collected and analyzed the data, and wrote the manuscript.

## Conflict of Interest Statement

The authors declare that the research was conducted in the absence of any commercial or financial relationships that could be construed as a potential conflict of interest.
